# Flammability Tests and Investigations of Properties of Lignin-Containing Polymer Composites Based on Acrylates

**DOI:** 10.3390/molecules25245947

**Published:** 2020-12-15

**Authors:** Beata Podkościelna, Krystyna Wnuczek, Marta Goliszek, Tomasz Klepka, Kamil Dziuba

**Affiliations:** 1 Department of Polymer Chemistry, Institute of Chemical Science, Faculty of Chemistry, Maria Curie-Sklodowska University, M. Curie-Sklodowska Sq. 3, 20-031 Lublin, Poland; krystyna.wnuczek@poczta.umcs.lublin.pl (K.W.); marta.goliszek@poczta.umcs.lublin.pl (M.G.); 2 Analytical Laboratory, Institute of Chemical Science, Faculty of Chemistry, Maria Curie-Sklodowska University, M. Curie-Sklodowska Sq. 3, 20-031 Lublin, Poland; 3 Department of Technology and Polymer Processing, Faculty of Mechanical Engineering, Lublin University of Technology, Nadbystrzycka 36, 20-618 Lublin, Poland; t.klepka@pollub.pl; 4 Department of Organic Chemistry, Institute of Chemical Science, Faculty of Chemistry, Maria Curie-Sklodowska University, M. Curie-Sklodowska Sq. 3, 20-031 Lublin, Poland; kamil.dziuba@poczta.umcs.lublin.pl

**Keywords:** flammability tests, kraft lignin, diethyl vinylphosphonate

## Abstract

In this paper flammability tests and detailed investigations of lignin-containing polymer composites’ properties are presented. Composites were obtained using bisphenol A glycerolate (1 glycerol/phenol) diacrylate (BPA.GDA), ethylene glycol dimethacrylate (EGDMA), and kraft lignin (lignin alkali, L) during UV curing. In order to evaluate the influence of lignin modification and the addition of flame retardant compounds on the thermal resistance of the obtained biocomposites, flammability tests have been conducted. After the modification with phosphoric acid (V) lignin, as well as diethyl vinylphosphonate, were used as flame retardant additives. The changes in the chemical structures (ATR-FTIR), as well as the influence of the different additives on the hardness, thermal (TG) and mechanical properties were discussed in detail. The samples after the flammability test were also studied to assess their thermal destruction.

## 1. Introduction

Flame retardant additives are intended to inhibit or to stop the polymer combustion process by acting either physically (cooling, fuel dilution, formation of a protective layer) or chemically (reaction in the solid or gaseous phase). As follows from industrial practice, phosphorous-containing flame retardants are widely used as an alternative to halogenated fire retardants, and may suppress fire in a polymer in two ways. The first mechanism proceeds through the thermal degradation of phosphorus flame retardants into phosphoric acid, which converts the polymer into carbon rich char, whereas the other one is based on migration into the vapor phase, quenching the radicals, or concurrently in both ways [1,2].

Phosphorus-based flame retardants combust more completely, especially when conversion of both polymer and flame retardant into char decreases the formation of gaseous phase degradation products. These volatile products can be hazardous to human health and, thus, the conversion to carbonaceous char is advisable.

**Figure 1:**
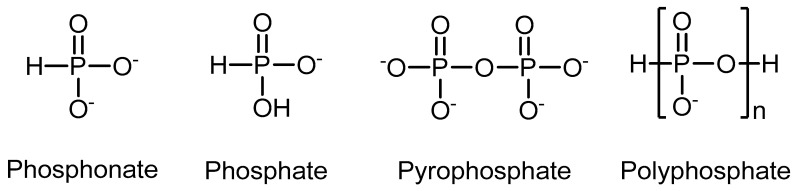
Chemical structure of the class of phosphorous compounds.

Depending on the molecular structure of the phosphorus moiety, different modes of flame retardancy and different type polymers can be targeted [3,4,5]. The class of phosphorous compounds includes two main types of flame retardants: ammonium polyphosphate and red phosphorus, as well as phosphonate, phosphate ester (pyrophosphate and polyphosphate), in which the phosphorus contents in the phosphorus containing flame retardants can start from a few percent to 100% (Figure 1) [6].

The combination of phosphorus chemistry and the effective use of biomass, e.g., lignocellulose, promotes the production of intrinsic flame-retardant materials.

Lignin is a highly abundant bio-polymeric material, being one of major components in the cell wall of woody plants, next to cellulose. Additionally, a large quantity of lignin is available every year from various pulping and paper industries [7,8,9,10]. Lignin is considered to be one of the most prospective and sustainable bio-resources for the development of the environmentally friendly polymer composites [11,12,13,14]. Due to its chemical structure and the presence of different functional groups it can provide additional functionality in composites such as a reinforcing agent, filler, stabilizer, compatibilizer, etc. [15,16,17]. Moreover, it could be effectively used as a carbon source in combination with other flame retardants to design the intumescent system for polymeric materials due to its high capability of char formation [18,19,20,21,22].

Phosphorylation has been proved to be an effective route for increasing the thermal stability of lignin [21]. Previous papers examined the lignin phosphorylation using diethyl phosphite [22,23], orthophosphoric acid [24], polyphosphoric acid [25], dihydrogen ammonium phosphate [26], phosphorus pentoxide [27], and DOPO (9,10-dihydro-9-oxa-10-phosphaphenanthrene-10-oxide) [28,29] and its derivatives are considered a suitable alternative to the halogenated flame retardants in the light of their effective extinguishing behavior in the gas phase and the condensed phase, as well as its environmental friendliness [22]. Combination of the phosphorus-based additives with the nitrogen-containing products, like melamine cyanurate, melamine phosphate, or melamine polyphosphate, exhibit high performance because of synergistic effects [30]. In turn, the bio-based flame retardants composed of lignin represent one of the most promising trends for new-generation flame retardants due to their sustainability and environmental benefits [31,32,33,34,35,36,37]. Moreover, the exploitation of other biomacromolecules as effective flame retardants for textiles was also studied [38].

In the paper synthesis, characteristics and flammability tests of biocomposites with kraft lignin are presented. For the study there were used two kinds of lignin: commercial kraft lignin and lignin after modification with phosphoric acid (V). Therefore, for the purpose of the flame retardant additives preparation, the effective synthetic route, which led to phosphorylated lignin, was developed in a simple process suitable for large-scale industrialization. The main component of the composites was an acrylic derivative of bisphenol A, the reagent commonly used for preparation of epoxy resins or polycarbonate plastics. The proposed materials belong to the group of hard and rigid composite materials for potential structural applications. In order to increase the flame-retardant effect, the diethyl vinylphosphonate was added. To evaluate the influence of lignin modification and addition of flame retardant compound on the thermal resistance of the obtained biocomposites, flammability tests were conducted. The changes in the chemical structures (ATR-FTIR), as well as the influence of the different additives on the hardness, thermal (TG) and mechanical properties are discussed in detail. In addition, after the flammability tests, the received carbons were also examined and the mechanism of decomposition was proposed.

## 2. Results and Discussion

### 2.1. Nuclear Magnetic Resonance Characterization of Lignin

**Figure 2:**
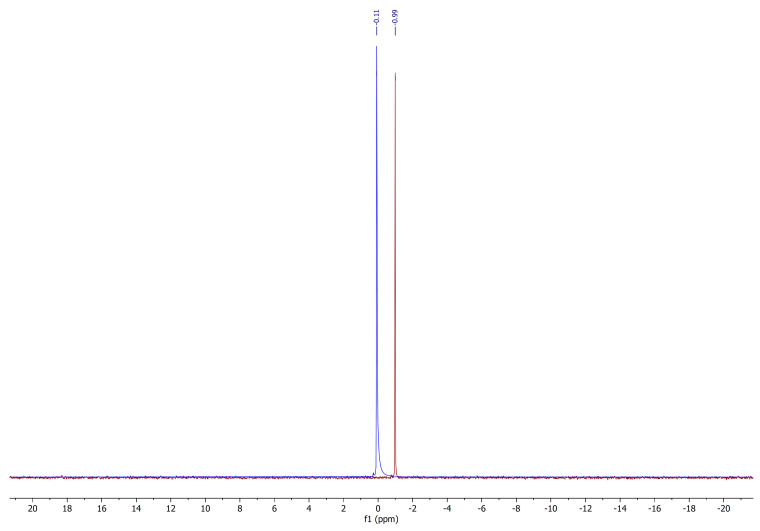
^31^P NMR spectra of H_3_PO_4_ and phosphorylated lignin in DMSO-*d_6_*.

^31^P NMR spectroscopy was applied in order to determine if the phosphorylation of the kraft lignin was effective. ^31^P NMR spectra in deuterated DMSO of phosphorylated lignin with ^1^H decoupling revealed one peak at −0.99 ppm. The comparison with H_3_PO_4_ (0.11 ppm) wide peak shows that the chemical shift of the phosphorylated lignin peak is close to the phosphoric acid but it is narrow shaped and shifted towards the higher field caused by the substitution of one hydroxyl group of the phosphoric acid by the kraft lignin moiety (Figure 2).

**Figure 3:**
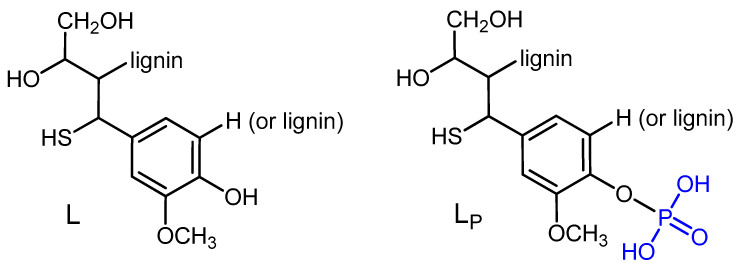
Phosphorylated guaiacyl units in the lignin structure.

No coupling with the protons from aliphatic skeleton is observed in the experiment without ^1^H decoupling, which indicates the presence of only one type of phosphorus selectively coupled with the aryl groups of the lignin guaiacyl units, which were also identified by ATR-FTIR spectroscopy (Figure 3).

**Figure 4:**
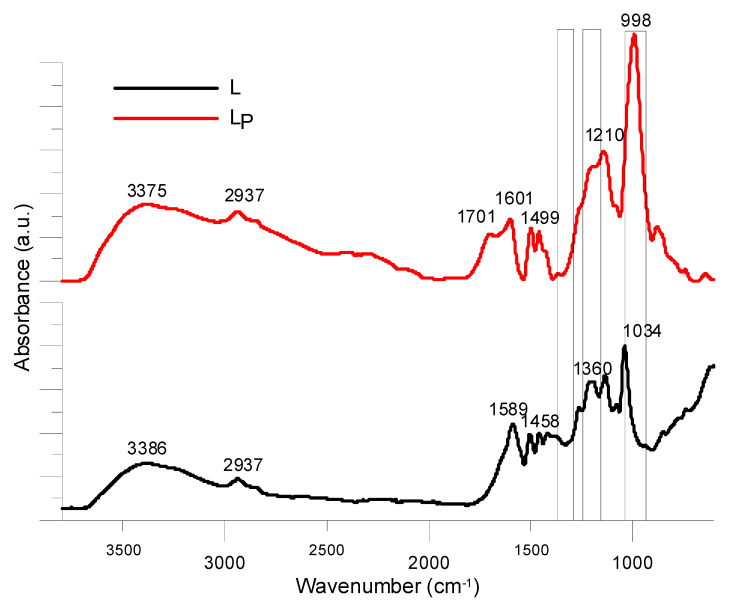
ATR-FTIR spectra of kraft lignin and phosphorylated lignin.

The kraft lignin ATR-FTIR spectrum shows classical absorption bands described in the literature. There is observed a wide band with the maximum at 3400 cm^−1^ from stretching vibrations of hydroxyl groups are involved in the aliphatic and aromatic chains and C–H vibrations of aliphatic skeleton are detected at 2935 and 2840 cm^−1^, respectively. Then, numerous absorption bands in the range of 2000 to 600 cm^−1^ are observed and disclose the complexity of the lignin structure. Depending on the source and the reference, several bands may be attributed to different lignin’s units (guaiacyl, syringyl) and then for specific chemical groups. The characterization of phosphorylated lignin was undertaken made by ATR-FTIR and its spectrum is compared to that of kraft lignin in Figure 4. It is shown that the phosphorylation process did not degrade the lignin structure and similar bands attributed to the aromatic units, hydroxyl and carbonyl groups were observed [39]. However, phosphorylated lignin exhibits a major change at the large and intense band centered at 1000 cm^−1^ attributed to the absorption of phosphate linkages present in the phosphate groups while the intensity of the phenolic OH band at 1364 cm^−1^ was dramatically reduced. Stretching of P = O (1200 cm^−1^) was also detected as the signals ratio 1210/1265 cm^−1^ (C-O stretching) in phosphorylated lignin is higher than that of kraft lignin.

### 2.2. Characterization of Cured Composites

**Figure 5:**
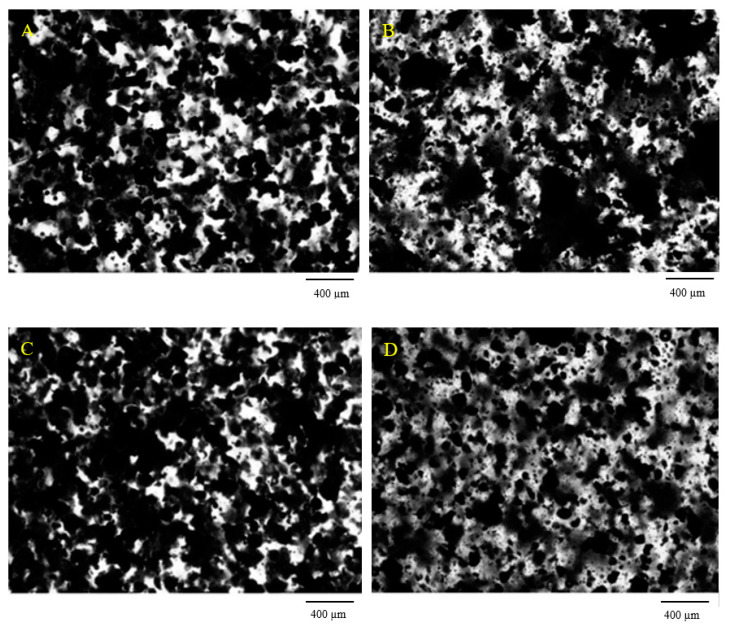
Photos of the synthesized biopolymers obtained using a Morphologi G3 optical microscope, magnification 2.5×, (**A**) 0%P + 5%L, (**B**) 0%P + 5%L_P_, (**C**) 2%P + 5%L, (**D**) 2%P + 5%L_P_.

In Figure 5 the photographs of the obtained composites with lignin are presented. Due to the high viscosity of BPA.GDA monomer, the lignin particles do not tend to fall to the bottom of the mould. For this reason, the relatively regular distribution of lignin particles in the materials is visible. As one can see, the samples look very similar and the size of lignin is about 50–200 µm. After modification, lignin has a greater tendency towards agglomeration which is reflected in the presence of larger lignin particles and lower transparency of the composite (samples: 0%P + 5%L_P_ and 2%P + 5%L_P_).

### 2.3. ATR-FTIR Analysis of Parent Composites

**Figure 6:**
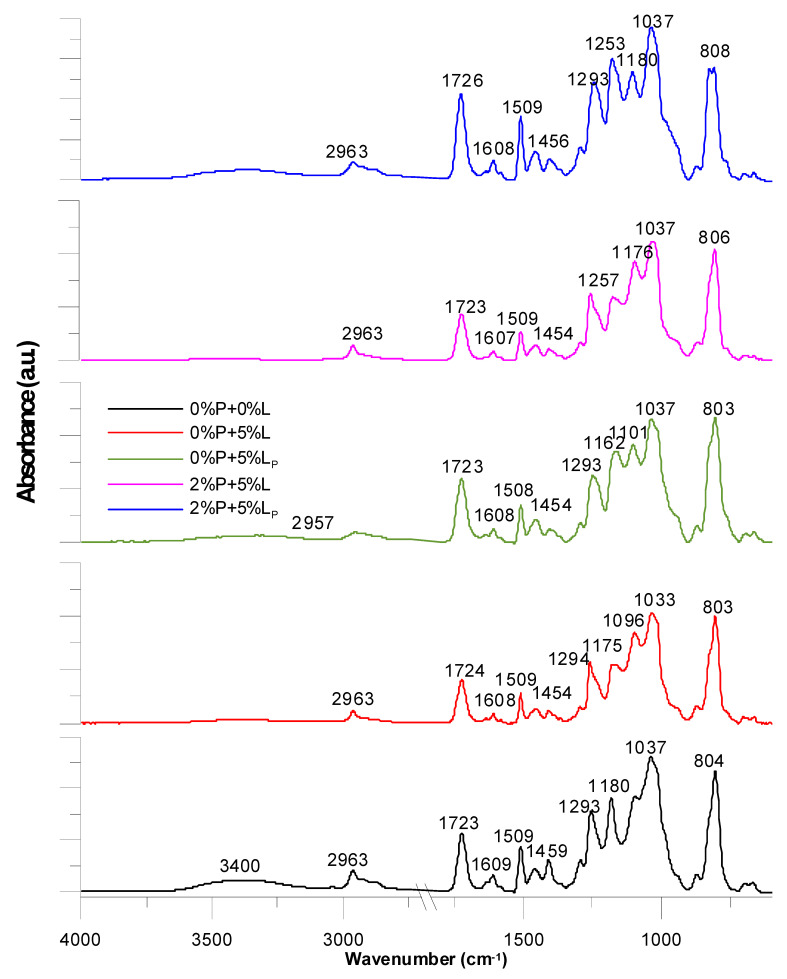
ATR-FTIR spectra of the studied composites.

The ATR-FTIR spectra are shown in Figure 6. The exact values of the wave numbers are also given in Table 1. Generally, the spectra of all obtained systems have a similar course. A hydroxyl group ranging from 3200–3500 cm^−1^ is visible for 0%P + 0%L copolymer. The broad absorption band with a maximum at 3400 cm^−1^ in the spectrum indicates the presence of –OH groups from the BPA.GDA monomer. In the modified composites this signal becomes weaker.

Another characteristic absorption band in the range of 2957–2963 cm^−1^ is derived from the stretching vibrations from the C-H aliphatic groups. This effect is visible for each composite. The characteristic signal is the peak in the range of 1723–1726 cm^−1^. It is derived from the carbonyl group that occurs in the EGDMA and DEVP structures as well as the phosphorylated lignin also. The peak at 1726 cm^−1^ (2%P + 5%Lp) is the most intensive for the copolymers containing the flame retardant agent and phosphoric acid lignin. Multiple bands ranging from 1609 cm^−1^ to 1454 cm^−1^ can be associated with the vibrations of C-H and C = C bonds related to the benzene rings and aromatic skeletons ones. They come from both the aromatic lignin part and BPA.GDA. The peaks around 1459–1406 cm^−1^ are from C-H deformation in the -CH_2_, -CH_3_ groups. The bands around 1293–1257 cm^−1^ could be attributed to C-O stretching. The signal at 806–804 was also associated with the C–C vibrations from the aromatic part. In summary, the addition of a flame retardant and lignin does not drastically change the course of the ATR-FTIR curves of the copolymers, however, it does affect the intensity of the signals.

### 2.4. Mechanical Properties

**Figure 7:**
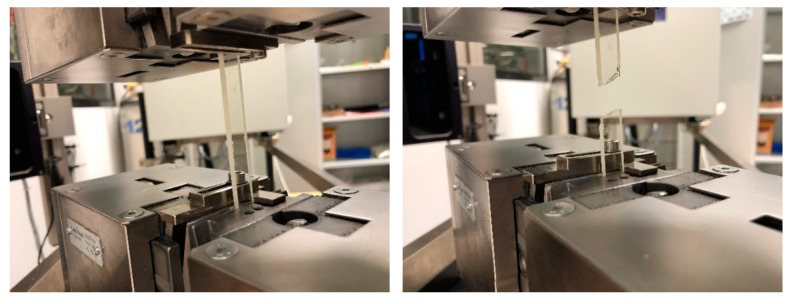
Exemplary samples during the mechanical tests.

The results of mechanical tests for the all studied materials are presented in Table 2 and Table 3. The static single-axis tensile test is the basic test method for structural strength materials [40,41]. During the test the dependence of the tensile force on the increase in the length of the specimen is recorded. In Figure 7 the exemplary sample (0%P + 0%L) during tensile stress is presented.

Another test of strength characteristics of products is bending which allows to determine the strength, modulus of elasticity, conventional yield strength, and bending deformation. It represents the highest stress that occurs in the material at the moment of damage [42,43,44].

As expected, the results of tests during the uniaxial stretching showed that the addition of lignin to all materials causes a decrease in the value of the elastic modulus (Young’s modulus), which is the effect of a decrease in tensile stress. The smallest changes are observed for the lignin modified with addition of flame retardant compound (DEVP). The largest change in the Young’s modulus is from 2910 MPa to 1440 MPa and the stress at break from 31.00 MPa to 8.00 MPa composition with a 5% addition of unmodified lignin. In compositions based on modified lignin, this biofiller interacts more strongly with the polymer network and exhibits higher breaking strength, comparable to the original polymer (0%P + 0%L) The data presented here show that mechanical properties of the studied compositions changed with the addition of eco-filler, but changes are smaller for the modified lignin [45]. The addition of a flame retardant in the form of a vinyl derivative does not significantly deteriorate the mechanical properties.

### 2.5. Thermogravimetric Analysis

**Figure 8:**
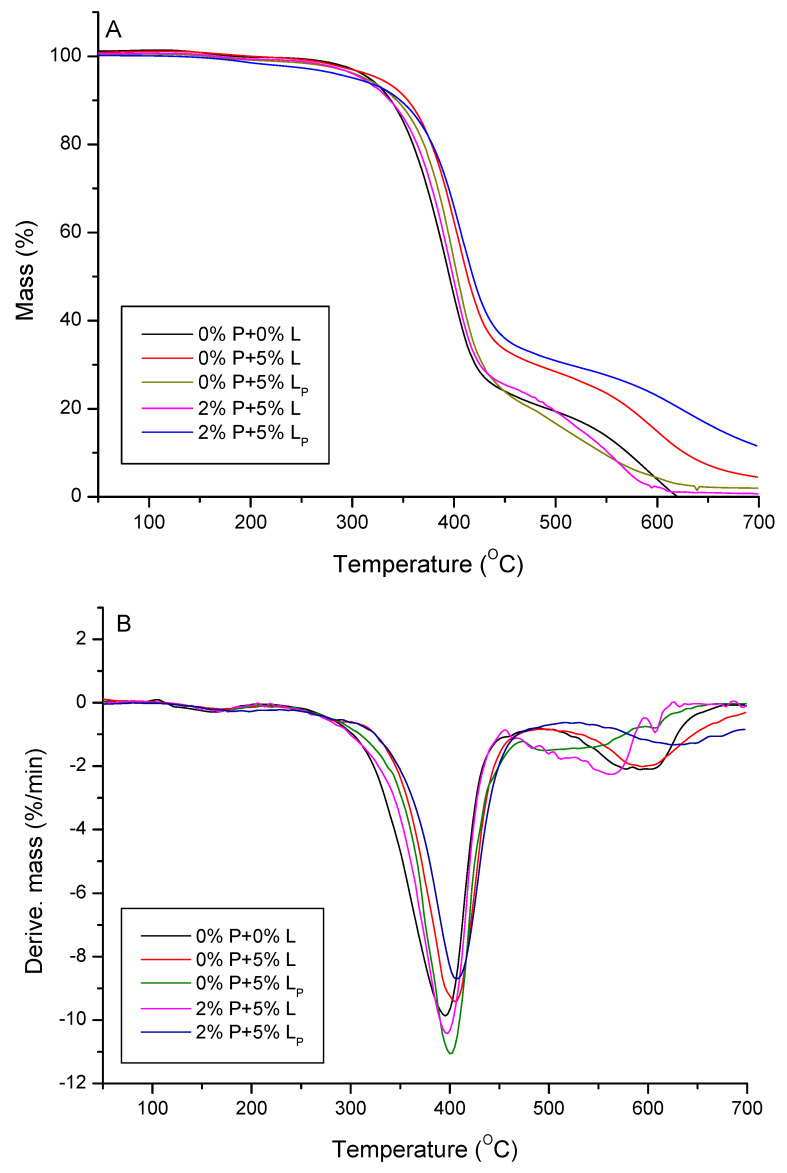
TG (**A**) and DTG (**B**) curves of the studied composites.

Thermal stability and degradation behavior of the obtained composites were investigated by means of thermogravimetry. The TG/DTG results of thermal decomposition process in the inert atmosphere of helium are presented in Figure 8 and Table 4. For the reference material without any additives (0% P + 0% L), as well as for the lignin-containing composites (0%P + 5%L, 0%P + 5%L_P_, 2% P + 5%L, 2%P + 5%L_P_), the TG curves have almost the same course and the initial decomposition temperature, corresponding to the temperature of 5% of mass loss is in the range 301–326 °C. It is related to the evaporation of residual solvents, moisture and unreacted monomers. Additionally, the polymer decomposition temperatures of 10% and 50% weight loss are reported. After an initial weight loss, the main weight loss starts at about 330–350 °C. The highest temperatures of 10% and 50% weight loss were reported for the 0%P + 5%L and 2% P + 5% L_P_ samples. The DTG curves for the studied polymers contain two main degradation steps. The first maximum decomposition peak (T_max1_) is observed in the range 396–407 °C. It is related to the main decomposition of polymer network fragments [46]. The highest temperatures of T_max1_ are reported for the 0%P + 5%L and 2%P + 5%L_P_ samples, the lowest ones for the reference material (0%P + 0%L) which clearly indicates improvement of thermal stability of studied materials as a result of additives. The other maximum decomposition peak (T_max2_) is observed in the range 521–621°C which is associated with the degradation of residual crosslinked parts of polymer structure. The highest value of T_max2_ was reported for the 2%P + 5%L_P_ sample. The residual mass (RM) evaluated at 700 °C is in the range 0.68–11.46%. The obtained results show that the studied materials are characterized by high thermal resistance additionally improved by the use of the additives.

### 2.6. Hardness of Composites

The Shore hardness values of the composites are presented in Table 5. The composites without lignin had the highest Shore hardness (80.5 °Sh). Analyzing the obtained results one can conclude that the addition of lignin reduces slightly the composites hardness by about 1–2 °Sh. No effect of fire retardant compound addition on the hardness of the composites is observed.

### 2.7. Flammability Tests

**Figure 9:**
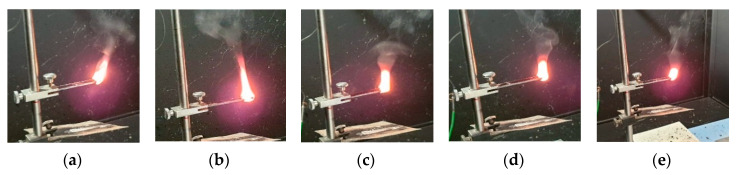
Photos of burning polymers during the tests. (**a**) 0%P + 0%L, (**b**) 0%P + 5%L, (**c**) 0%P + 5%L_P_, (**d**) 2%P + 5%L, (**e**) 2%P + 5%L_P_.

The flammability tests were taken out in a horizontal burning test. Figure 9 shows the burning compositions. The samples were burning for 60 s (30 s over a burner and then self-burning for 30 s). Sample No. 1 (without additives) and sample No. 2 (with lignin kraft) burn the most which manifests in the brightest and greatest flame. The addition of modified H_3_PO_4_ lignin and DEVP reduces the flame height and intensity (samples 3–5).

The studies showed that the addition of natural lignin filler did not increase the burning rate of composites compared to the original material without additives (BPA.GDA-*co*-EGDMA). When comparing the analyzed composites, the differences appearing in the course of the combustion process can be noticed. All the tested samples burnt with a bright flame, after burning the samples were charred with no visible blisters or cavities. Nevertheless, the additives affected the appearance, shape and size of the flame, the temperature in the center area of the burning sample, and the rate at which the fire was absorbed by the sample section.

**Figure 10:**
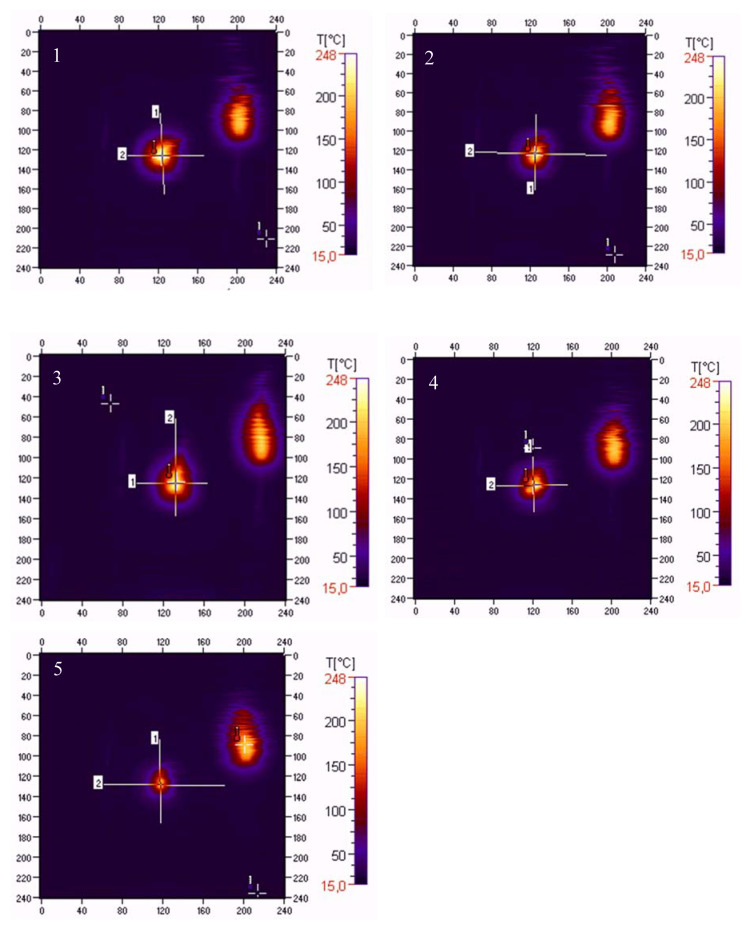
Photos of the samples made using a thermographic camera (**1**) 0%P + 0%L, (**2**) 0%P + 5%L, (**3**) 0%P + 5%LP, (**4**) 2%P + 5%L, (**5**) 2%P + 5%LP).

**Figure 11:**
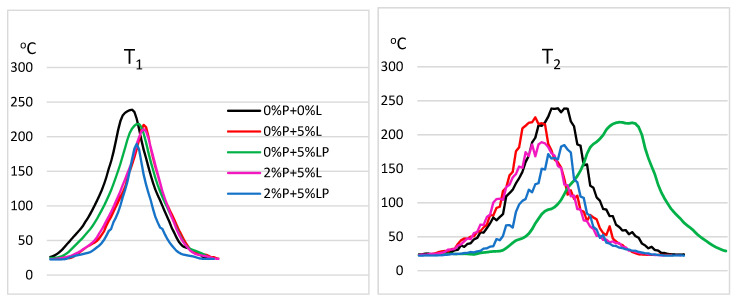
Flame temperature curves for the distribution of measuring points along the horizontal (1) and vertical (2) curves (T_1_ and T_2_) from the thermographic camera.

Figure 10 shows the photos of the samples made using a thermographic camera. During the free burning of the sample, a recording photo was taken on the thermogram, two lines were marked along which the values of temperature changes during the sample burning were determined for the horizontal and vertical systems, respectively. Two specific smoking areas marked with symbols 1 and 2, where the minimum and maximum burning temperatures can be distinguished (Table 6). The highest temperature values were recorded for the sample without additives at 238 °C (0%P + 0%L) and the lowest in the range of 184–189 °C for the modified DEVP and L_P_ sample (2%P + 5%L_P_). Additionally, flame temperature curves for the distribution of measuring points along the horizontal and vertical curves from the thermographic camera are presented in Figure 11.

**Figure 12:**
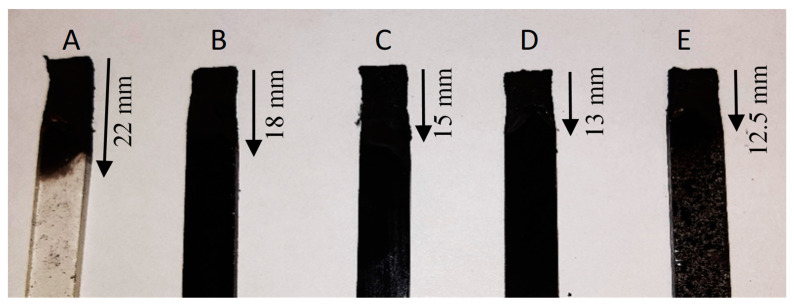
The samples after the burning test (**A**) 0%P + 0%L, (**B**) 0%P + 5%L, (**C**) 0%P + 5%L_P_, (**D**) 2%P + 5%L, (**E**) 2%P + 5%L_P_).

Figure 12 shows the samples after the burning test. The polymer without additives burnt the fastest (22 mm in 60 s) and the slowest (by 57%) was the composite No. 5 (2%P + 5%L_P_). The samples burnt only on the surface and maintained their shape. The composite flammability tests show clearly that the addition of a flame retardant and lignin affects the burning behavior of the composites. The composite without additives burnt the fastest and most intensively. The addition of lignin itself caused a slight delay in the burning process. The modification of lignin did not drastically affect its flame retardancy but it certainly slowed down this process. The greatest delay was found for the sample with DEVP and L_P_, but the sample was burning, however, the temperature of this process was lower, the flame had a less intense shape and had a shorter burning distance. In summary, the research should be carried on with the use of more modified lignin and other flame retardants.

### 2.8. Characterization of Composites after Flammability Tests

**Figure 13:**
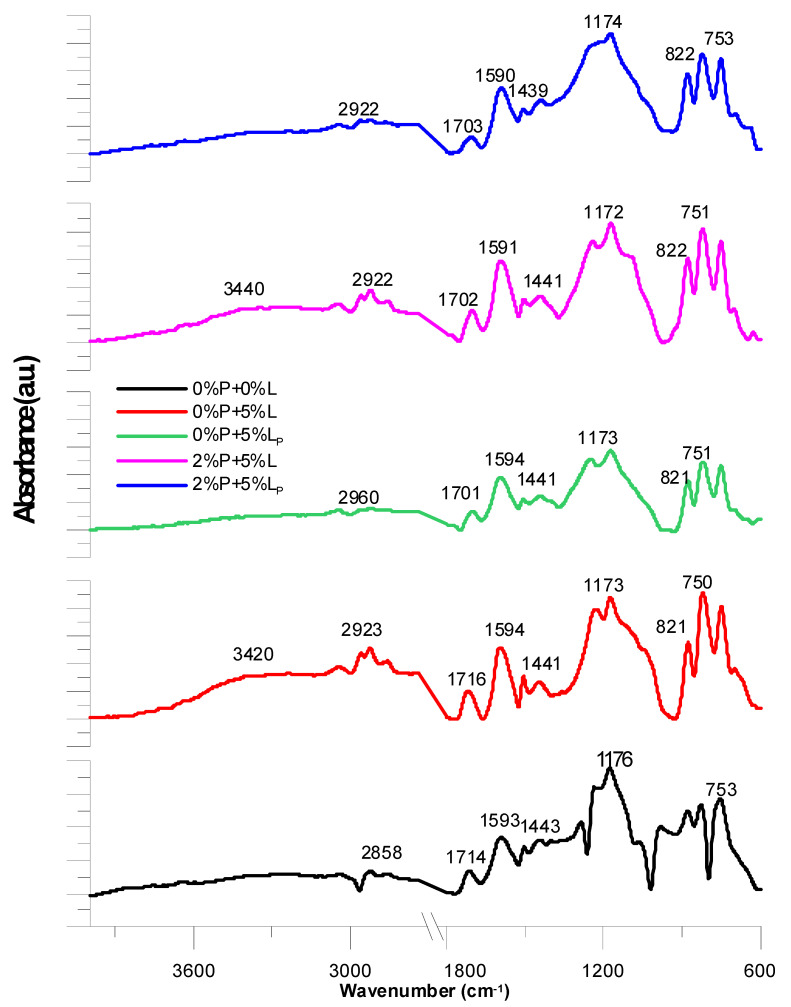
ATR-FTIR analysis of the composites after the flame tests.

The composite material after the flame tests was also subjected to ATR-FTIR analysis. The obtained spectra are shown in Figure 13. The flattened signal for the hydroxyl group is visible only for the copolymers 0%P + 5%L and 2%P + 5%L. It probably comes from unmodified kraft lignin due to free hydroxyl and phenolic groups present in its structure. All spectra are similar to each other. The combustion process was conducted for only 60 s, therefore, in the spectra, signals from uncharred fragments of compositions are also visible. Presumably the composites underwent surface carbonization (incomplete combustion). This is evidenced by the signals such an absorption bands characteristics of the C=O groups, C–O groups, C–H bonds. Signals from the aromatic part of the composite in each spectrum are also observed. The resulting numerous aromatic signals testify to possible cyclization of linear fragments of chains. There was also an increase in the aromatic ring vibration intensity signals in the range 1500–1610 cm^−1^ coming from the C–H groups, while the signals in the range 1500–1610 cm^−1^ correspond to the stretching vibrations of the C=C group. Numerous vibrations in the range of 1450–1610 cm^−1^ indicate skeletal vibrations of the ring.

**Figure 14:**
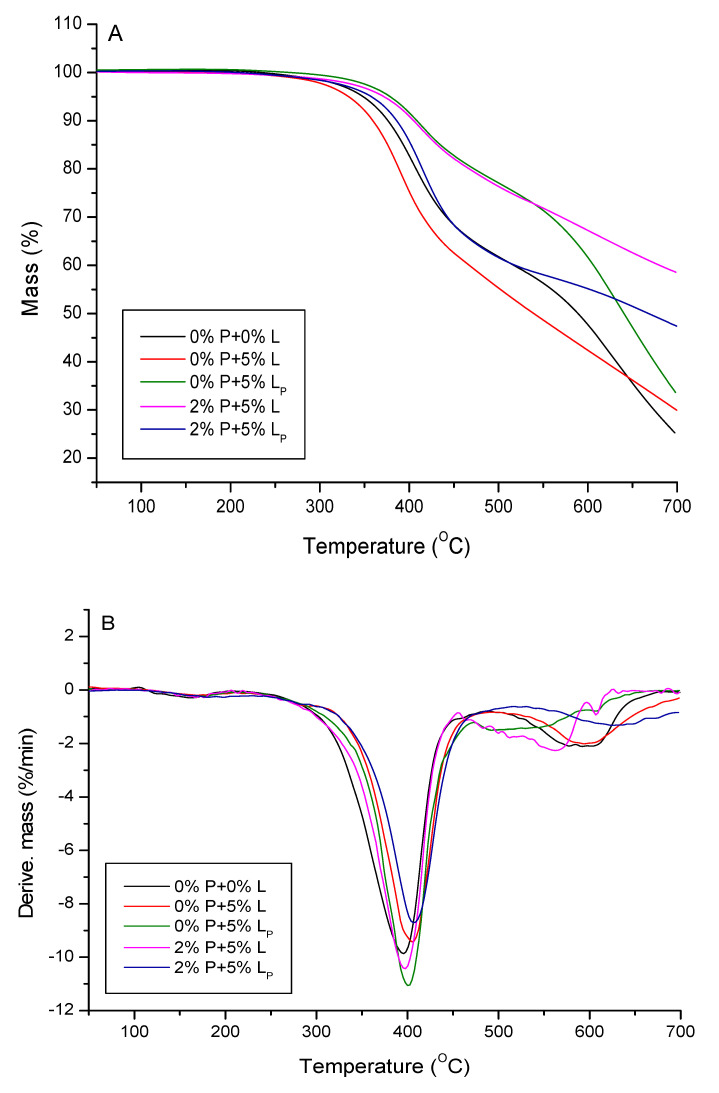
TG (**A**) and DTG (**B**) curves after the flame tests.

Additionally, thermal behavior of the samples after the flame tests was studied by the thermogravimetric analysis. The TG/DTG results are presented in Figure 14 and Table 7. According to the reported temperatures of 5%, 10%, and 50% weight loss, the materials with L_P_ are characterized by higher thermal resistance in comparison to the reference material without the additives (0%P + 0%L). Based on the DTG curves the thermal degradation process of the 0%P + 0%L and 0%P + 5%L_P_ samples proceeds in two steps (T_max1_ and T_max2_), whereas for the other samples one peak in observed on the DTG curves (T_max1_). The values of RM are in the range 28.84–58.49%. A significant change in residual mass compared to the initial samples (0.68–11.46%) is visible. The addition of modified lignin and DEVP expressively increases of the mass residue. These additives can be used as modifiers to obtaining carbons, e.g., for sorption applications.

**Figure 15:**
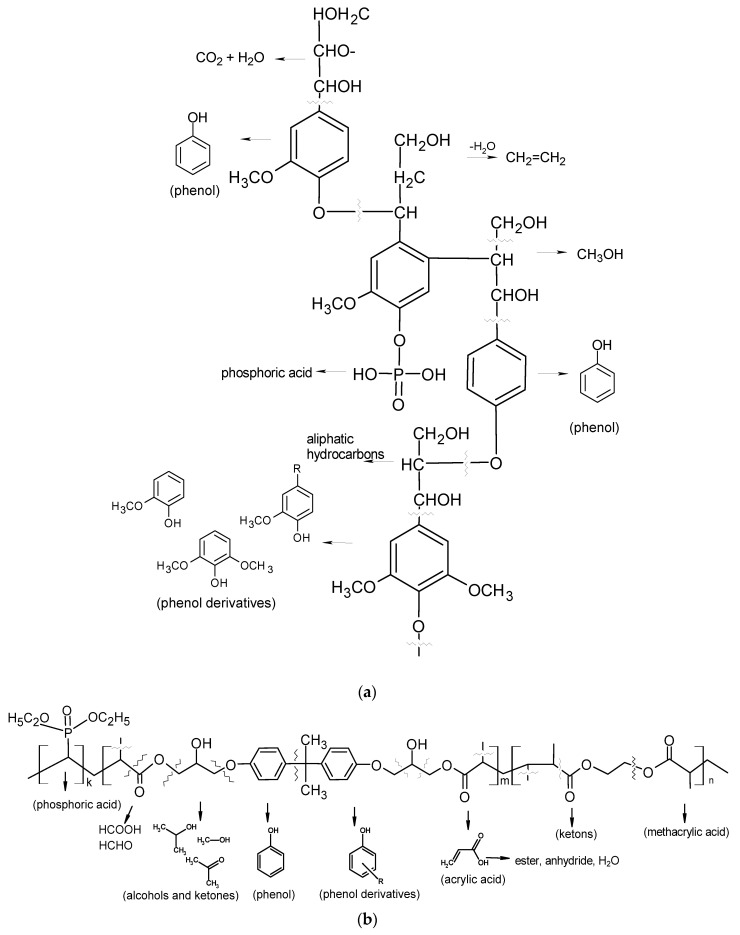
Proposed mechanisms of modified lignin (**a**) and copolymers (**b**) fragmentation under heating.

In Figure 15 the mechanisms of the possible composite network fragmentation and thermal decomposition products are presented. The proposed mechanisms are based on our earlier research, analysis of the gaseous products of decomposition for polymeric microspheres with lignin and BPA.GDA [47].

## 3. Materials and Method

### 3.1. Materials and Chemicals

Bisphenol A glycerolate (1 glycerol/phenol) diacrylate (BPA.GDA), ethylene glycol dimethacrylate (EGDMA), kraft lignin (lignin alkali, L) sulfur content <3.6%, pH 10–11, diethyl vinylphosphonate (DEVP), and 2-dimethoxy-2-phenylacetophenone (Irgacure 651, IQ) were from Sigma-Aldrich (Darmstandt, Germany). Phosphoric acid (H_3_PO_4_, 85%) was obtained from Avantor Performance Materials Poland S.A. (Gliwice, Poland). Purified water was provided by Millipore UMCS (Lublin, Poland).

### 3.2. Modification of Kraft Lignin

Into a 100 (mL) reaction flask equipped with a mechanical stirrer and a thermometer 10 g of kraft lignin and 30 mL of H_3_PO_4_ (85%) were added. Initially the reaction proceeded at room temperature, after 12 h the mixture was heated to 50 °C, the final temperature was maintained for 30 min. The modified lignin was filtered off and washed with distilled water to obtain a neutral pH.

### 3.3. Synthesis of Composites

The BPA.GDA with EGDMA copolymer in the presence of UV initiator (Irgacure 651, IQ) and kraft lignin, or modified kraft lignin with H_3_PO_4_ was obtained. Firstly, the appropriate amounts of BPA.GDA and EGDMA (7:3 wt%) were added into the glass vessel and transferred to the heating chamber to deaerate the samples (60 °C). Next, a suitable amount of kraft lignin or modified kraft lignin or DEVP was transferred to the mixture. Finally, the UV initiator was added to the liquid composition (2 wt%). The experimental parameters of the syntheses are presented in Table 8.

**Figure 16:**
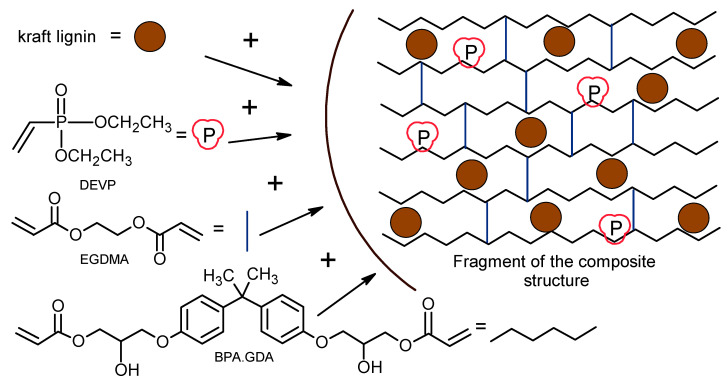
Chemical structure of monomers and the exemplary fragment of composites structure.

The liquid compositions containing the initiator, monomers or/and lignin or/and DEVP were placed inside the irradiation chamber where they were exposed (30 min) to UV light with two mercury lamps of 500 W. The exemplary fragment of the composite structure is presented in Figure 16.

The application of crosslinking monomers (EGDMA and BPA.GDA) results to obtaining rigid polymer network. The fire retardant (DEVP) possess vinyl groups in its construction and can copolymerize with monomers forming the polymer chain. Lignin is used as an ecofiller, but due to its chemical structure (presence of polar groups), it can also interact with carbonyl (C=O) and hydroxyl (-OH) groups existing in monomers. Lignin can affect with polymer chains by weaker intermolecular interactions and hydrogen bonds which contribute to its stronger incorporation into the polymeric network.

### 3.4. Characterization Methods

The attenuated total reflection (ATR) was recorded using the infrared Fourier transform spectroscopy on a TENSOR 27, Bruker spectrometer, equipped with a diamond crystal (Ettlingen, Germany). The spectra were recorded in the range of 600–4000 cm^−1^ with 32 scans per spectrum at a resolution of 4 cm^−1^.

The NMR spectra were recorded on a Bruker AV500 (1H 500 MHz, 31P 202 MHz, 13C NMR 126 MHz) spectrometer (Coventry, United Kingdom). All spectra were obtained in deuterated the DMSO solutions, unless mentioned otherwise, and the chemical shifts (d) are expressed in ppm using the internal reference to TMS with the solvent as an internal indicator and the external reference to 85% H_3_PO_4_ in D_2_O for ^31^P. The coupling constants (J) are given in Hz. The abbreviations of signal patterns are as follows: s, singlet; d, doublet; t, triplet; q, quartet; m, multiplet; b, broad.

The fragments of the solid composites were studied using a Morphologi G3 optical microscope (Malvern, Great Britain).

Thermal analysis was conducted using a STA 449 Jupiter F1, Netzsch (Selb, Germany). The samples were heated from 30–800 °C at a rate of 10 °C min^−1^ in a dynamic atmosphere of helium (25 cm^3^ min^−1^). The sensor thermocouple of S TG–DSC type was used with an empty Al_2_O_3_ crucible as a reference.

The hardness of the materials was measured by the Shore D method using a 7206/H04 analog hardness testing apparatus, Zwick (Ulm, Germany) at 23 °C. Readings were taken after 15 s.

The samples in the form of specimens (2 mm × 10 mm × 60 mm) were subjected to the strength tests during the uniaxial tensile strength. The tests were taken according to EN-ISO 527, the test speed of 50 mm/min at 23 °C. In order to examine the bending strength, a test was made with the used the three-point bending tests (EN-ISO 170, ASTM D-790 standards). All tests were taken using a Zwick/Roell Z010 universal tensile-testing machine (Ulm, Germany). The test specimens were cut from the pressed sheet form.

The flammability tests were taken in the laboratory of Department of Technology and Polymer Processing, Lublin University of Technology. The flammability testing device was equipped with a combustion chamber, ventilation system, and a thermal imaging camera. During the test, the samples were fixed to a tripod, then the burner (methane) was brought closer to the sample for 30 s, at an angle of 45°. After that time, the samples burnt freely for another 30 s. The flammability tests were taken in a horizontal burning test according to the PN-EN 60695-11-10—method A. During the burning process, observations were made using a V-20 thermovision camera model ER005-25 (Vigo System, Ożarów Mazowiecki, Poland) in the temperature measurement range from −10 to 500 °C. During the tests two photos were taken for each sample—after removing the burner and after 15 s of free burning.

## 4. Conclusions

New cross-linked composites based on bisphenol A glycerolate (1 glycerol/phenol) diacrylate (BPA.GDA) and ethylene glycol dimethacrylate (EGDMA) with the functional additives were successfully obtained during the UV-curing methodology. The first additive to the composites was lignin in two varieties, the original kraft lignin and its modification with phosphoric acid (V). The phosphorylation reaction of lignin was confirmed by the ^31^P NMR and ATR-FTIR spectroscopy. The other modifier was diethyl vinylphosphonate, (DEVP). The influence of lignin modification and the addition of flame retardant compound on the thermal resistance of the obtained biocomposites was evaluated. According to the flammability tests the addition of a flame retardant and lignin affects the burning behaviors of the composites. The materials without the additives burnt the fastest and most intensively. The addition of lignin itself caused a slight delay in the burning operation. The lignin modification definitely slowed down this process. Based on the thermogravimetric analysis the studied materials are characterized by high thermal resistance which is improved by the use of the additives. After the flame tests the samples were also subjected to ATR-FTIR and thermogravimetry. As stated, composites were only partially burned, therefore, on the spectrum the signals characteristic of the C=O and C–O groups, as well as an increase in the aromatic ring vibration intensity signals are observed. The TG research indicates that the addition of modified lignin and DEVP to the composites significantly increases the mass residue (up to 58%). Thus, these modifications can be used as modifiers to obtaining carbon sorbents with high efficiency.

## Figures and Tables

**Table 1:** Wavenumbers (1/cm) of characteristic bands visible in the Fourier transform infrared—attenuated total reflectance spectra.

Material	C–H Aliph.	C–H Arom.	C = C Arom.	C-C	C–O	C = O	–OH
Lignin	2937	1504 1458	1589	1040	1210	-	3386
Lignin + H_3_PO_4_	2939	1499 1460	1601	993	1143	1701	3375
Composites							
0%P + 0%L	2963 1293	1459 1408	1609 1509	1254 1180	1293	1723	3400
0%P + 5%L	2963 1263	1454 1406	1608 1509	1294 1175	1294	1724	-
0%P + 5%L_P_	2957 1101	1454 1403	1608 1508	1293 1162	1293	1723	-
2%P + 5%L	2963	1454 1407	1607 1509	1257 1176	1257	1723	-
2%P + 5%L_P_	2963	1456 1406	1608 1509	1253 1180	1293	1726	3344
Composites after burning tests							
0%P + 0%L	2858 1295	1443	1593	1176 828	753	1714	-
0%P + 5%L	2923 2858	1441	1594	1175 821	750	1716	3420
0%P + 5%L_P_	2960	1441	1594	1173 821	751	1701	-
2%P + 5%L	2922	1441	1591	1172 822	751	1702	3440
2%P + 5%L_P_	2922	1439	1590	1174 822	753	1703	-

**Table 2:** Results of mechanical tensile stress.

Composite	Stress at Break (MPa)	Relative Elongation at Break (%)	Young’s Modulus (MPa)
0%P + 0%L	31.00	1.10	2910
0%P + 5%L	8.00	0.34	1440
0%P + 5%L_P_	8.83	0.37	1450
2%P + 5%L	10.10	0.65	2450
2%P + 5%L_P_	9.36	0.37	2470

**Table 3:** Results of mechanical bending tests.

Composites	Stress during Bending (MPa)	Bending Elongation (%)	Young Modulus (MPa)
0%P + 0%L	49.85	1.20	3890
0%P + 5%L	36.00	0.85	3025
0%P + 5%L_P_	32.05	1.00	3895
2%P + 5%L	27.30	0.90	2950
2%P + 5%L_P_	48.60	1.00	4560

**Table 4:** TG/DTG data.

Composite	T_5%_ (°C)	T_10%_ (°C)	T_50%_ (°C)	T_max1_ (°C)	T_max2_ (°C)	RM (%) (at 700 °C)
0%P + 0%L	317	338	396	396	611	3.14
0%P + 5%L	326	355	413	406	597	4.45
0%P + 5%L_P_	313	344	403	401	521	1.98
2%P + 5%L	310	337	400	397	563	0.68
2%P + 5%L_P_	301	347	419	407	621	11.46

**Table 5:** Shore hardness of the composites.

Composite	Shore Hardness Mean (°Sh)
0%P + 0%L	80.5 ± 0.5
0%P + 5%L	78.6 ± 0.5
0%P + 5%L_P_	78.0 ± 0.5
2%P + 5%L	78.1 ± 0.5
2%P + 5%L_P_	78.4 ± 0.5

**Table 6:** The data from the thermographic camera.

Composite	Temperature (°C)			
	T_1_		T_2_	
	Min.	Max.	Min.	Max.
0%P + 0%L	23.92	238.92	23.45	238.92
0%P + 5%L	23.19	225.81	22.90	217.01
0%P + 5%L_P_	24.63	218.21	23.29	218.53
2%P + 5%L	22.93	189.11	23.07	210.87
2%P + 5%L_P_	22.12	184.83	22.17	189.67

**Table 7:** TG/DTG data after the flame tests.

Composite	T_5%_ (°C)	T_10%_ (°C)	T_50%_ (°C)	T_max1_ (°C)	T_max2_ (°C)	RM (% at 700)
0%P + 0%L	349	376	589	405	621	28.84
0%P + 5%L	332	359	540	389	-	29.98
0%P + 5%L_P_	379	409	650	411	623	33.29
2%P + 5%L	372	405	-	413	-	58.49
2%P + 5%L_P_	358	386	668	414	-	47.40

**Table 8:** Experimental parameters of the synthesis.

No.	Composite	BPA.GDA	EGDMA	DEVP	L	L_P_	IQ
		(g)					
1	0%P + 0%L	5	2.143	0	0	0	0.143
2	0%P + 5%L	5	2.143	0	0.364	0	0.150
3	0%P + 5%L_P_	5	2.143	0	0	0.364	0.150
4	2%P + 5%L	5	2.143	0.143	0.364	0	0.150
5	2%P + 5%L_P_	5	2.143	0.143	0	0.364	0.150

Where: DEVP—diethyl vinylphosphonate; L-kraft lignin; L_P_-kraft lignin after modification with H_3_PO_4_, IQ—Irgacure 651.
